# Global patterns of disease progression in inflammatory bowel disease: a comprehensive synthesis of contemporary population-based cohorts

**DOI:** 10.1093/gastro/goag013

**Published:** 2026-02-20

**Authors:** Beatriz Gros, Carlos Frutos, Beatriz Carrillo Cubero, María Gómez, Nikolas Plevris, Charlie W Lees

**Affiliations:** Gastroenterology and Hepatology Department, Reina Sofía University Hospital, Avenida Menéndez Pidal, Cordoba 14005, Spain; Maimónides Institute of Biomedical Research, IMIBIC, Avenida Menéndez Pidal, Córdoba, 14005, Spain; Biomedical Research Center in Hepatic and Digestive Disease, CIBEREHD, C/ Monforte de Lemos 3-5, Madrid, 28029, Spain; Gastroenterology and Hepatology Department, Reina Sofía University Hospital, Avenida Menéndez Pidal, Cordoba 14005, Spain; Gastroenterology and Hepatology Department, Reina Sofía University Hospital, Avenida Menéndez Pidal, Cordoba 14005, Spain; Gastroenterology and Hepatology Department, Reina Sofía University Hospital, Avenida Menéndez Pidal, Cordoba 14005, Spain; Edinburgh IBD Unit, Western General Hospital, Crewe Road South, Edinburgh, EH4 2XU, United Kingdom; Edinburgh IBD Unit, Western General Hospital, Crewe Road South, Edinburgh, EH4 2XU, United Kingdom; Centre for Genomic and Experimental Medicine, Institute of Genetics and Cancer, University of Edinburgh, Crewe Road South, Edinburgh, EH4 2XU, United Kingdom

**Keywords:** inflammatory bowel disease, Crohn’s disease, ulcerative colitis, colon cancer, extraintestinal manifestations, disease progression

## Abstract

Inflammatory bowel diseases (IBD), including Crohn’s disease (CD) and ulcerative colitis (UC), follow heterogeneous clinical trajectories. Although therapeutic options have expanded substantially over the past two decades, the extent to which modern treatment modifies long-term structural outcomes remains uncertain. We performed a targeted review focusing on high-quality population-based inception cohorts and large registries that report long-term outcomes in adult- and pediatric-onset IBD. Outcomes of interest included phenotype or extent progression, surgery, extraintestinal manifestations (EIMs), and colorectal neoplasia. CD consistently emerged as the more structurally progressive condition. Approximately one third of adults’ progress from inflammatory to stricturing or penetrating disease within 5 years, and around half do so over longer follow-up. Perianal disease develops in 10%–20% of patients, with higher rates in pediatric-onset CD. Despite declines in surgical rates in the biologic era, intestinal resection remains frequent. In UC, proximal extension is the dominant progression pattern, affecting roughly one third of patients with limited disease over the first decade; pediatric UC shows even higher extension rates. Colectomy risks have markedly decreased in contemporary cohorts, and colorectal cancer incidence has declined compared with historical estimates, reflecting improved inflammation control and surveillance. Across IBD, EIMs occur in approximately one quarter of patients and cluster with extensive colonic involvement and higher systemic inflammatory burden. Population-based evidence reveals that IBD remains progressive in a substantial subset of patients, with notable differences between CD and UC and between adult and pediatric disease. Declining surgical and colorectal cancer rates suggest a measurable therapeutic era effect, supporting the importance of early, sustained inflammation control. However, high-quality prospective disease-modification trials are still needed to further characterize how current strategies can durably alter the natural history of IBD.

## Introduction

Inflammatory bowel diseases (IBD), including Crohn’s disease (CD) and ulcerative colitis (UC), are chronic, immune-mediated diseases with a highly heterogeneous course. Although many patients achieve periods of clinical remission, a substantial proportion accumulate structural and functional damage over time.

Historically, CD has been considered a progressive disease, in which an initially inflammatory phenotype (B1-Montreal classification) frequently evolves into stricturing or penetrating complications (B2/B3), often requiring intestinal resection [1]. UC was traditionally viewed as a disease confined to the mucosal layer with a relatively benign course; however, population-based studies have shown that proximal extension of colitis, colectomy and extraintestinal manifestations (EIMs) are not uncommon, and that long-standing extensive colitis carries an increased risk of colorectal cancer (CRC) [2]. In addition, thanks to the increased usage of intestinal ultrasound, it has become clearer that UC is also a transmural disease [3].

The epidemiology and natural history of IBD have been described in population-based cohorts and systematic reviews over the last two decades. Burisch and Munkholm summarized how incidence, prevalence, surgery and cancer risks have evolved, highlighting that while therapeutic options have expanded, the extent to which modern therapy alters long-term disease course remains uncertain [4].

Large population-based inception cohorts, such as Epi-IBD, have generated robust and granular outcome data for both CD and UC in the modern immunomodulator and biologic era [5–7]. Together, these findings underpin the emerging concept of disease modification and the still-unresolved question of whether we can truly alter the natural history of IBD [8].

In this context, clinicians and researchers increasingly need a concise yet comprehensive synthesis of long-term progression patterns in both UC and CD, distinguishing adult and pediatric-onset disease. The objective of this review is to analyze and quantify the progression of CD and UC across four critical domains: the evolution of disease extent or phenotype, the development of new EIMs, the risk of CRC or dysplasia, and the cumulative need for surgery or colectomy. Special attention is paid to stratifying the results between adult and pediatric populations from population-based and inception cohorts, as well as the impact of therapeutic eras (pre-biologic vs biologic).

## Methods

Given the breadth of outcomes (colectomy, abdominal surgery, extension, EIMs, dysplasia, and CRC) and the heterogeneity of reporting across studies, we conducted a targeted literature review. Initial exploratory searches using MeSH terms in PubMed and Embase on 7 September 2025 returned more than 10,000 references. Due to the volume and overlap across outcomes, we adopted a targeted strategy centered on high-quality population-based inception cohorts and large registries. Additional studies were identified through forward and backward citation tracking. Search strategy and studies identified are presented in Supplementary Material.

We searched for the following outcomes:

1. Phenotypic (CD) or extent (UC) Progression: Quantification of the transition from inflammatory (B1) to stricturing (B2) or penetrating (B3) behavior in CD, and proximal extension (E1/E2 to E3) in UC.
2. Surgery rates: Cumulative incidence of intestinal resection in CD and colectomy in UC.
3. Extraintestinal manifestations: Incidence of new EIMs.
4. New occurrence of dysplasia and neoplasia: Risk of CRC/dysplasia.

Estimates are presented as approximate ranges of cumulative incidence over defined timepoints (typically 5, 10, or 20 years) and are contextualized by identifying major clinical determinants (such as age at onset, disease location/extent, and smoking) and the impact of the therapeutic era.

## Results

### Global patterns of progression in IBD

Evidence in this review is primarily derived from population-based inception cohorts (including Epi-IBD), nationwide Scandinavian cohorts, and large registries, complemented by systematic reviews and meta-analyses; therefore, estimates are reported as ranges rather than pooled effect sizes.

Across the synthesis of evidence, CD consistently emerged as the more structurally progressive condition when compared with UC. In adult CD, approximately one third of patients’ progress from an inflammatory phenotype (B1) to stricturing or penetrating behavior (B2/B3) within 5 years, and around half do so over a 20-year horizon based on estimates from population-based cohorts and inception studies [1, 5, 9]. Perianal disease estimates come from a mix of population-based cohorts and nationwide studies, with long-term cumulative risks ranging from approximately 10% to 20% and reaching higher values in extended follow-up cohorts (Table 1) [25, 26, 61].

**Table 1:** Benchmark estimates of major disease progression outcomes in adult- and pediatric-onset inflammatory bowel disease.

Outcome	Adults	Pediatric-onset
CD: Behavior progression (B1→B2/B3)	≈33% at 5 years; ≈50% by 20 years [1, 5, 9–11]	≈34% at 5 years vs 16% in adults; ≈41% long-term (any complication) [12–14]
CD: Intestinal resection	Historic 61% at 10 years, 82% at 20 years. Contemporary: 18%–35% at 5 years [5, 15–17].	13%–34% at 5 years; VEOIBD: ≈3% at 1 year, 12% at 3 years, 15% at 5 years [18–24].
CD: Perianal disease	8%–14% at 1 year; 17%–22% at 5 years; 18%–20% at 10 years; 20%–25% at 20 years [25–30].	28% at 10 years and 45% at 30 years [31].
CD: Colorectal cancer (colonic CD)	Increased risk; absolute incidence low; RR ≈9 when >50% colon involved for >10 years [32–35].	Very sparse data; risk likely low but uncertain.
EIMS in CD	35% long-term [36–38] - PSC <1% [39] - Skin 19% [40] - Joint 12.9% at 10 years [36]	Similar magnitude; limited pediatric-only data [41].
UC: Proximal extension from limited disease	≈33% at 7 years; ≈13% 5-year pooled risk [42–44].	30%–49% children with limited disease extend proximally over 5-10 years [24, 45, 46].
UC: Colectomy	Historic 24% at 10 years; Contemporary: 4.4% at 1 year, 10.1% at 5 years and 14.6% at 10 years [47, 48].	3%–6% at 1 year; 9%–14% at 5 years [49, 50]; VEOIBD 11%–14% at 5 years [49, 51–53].
CRC in UC	≈0.8% at 10 years; 1.15% at 15 years; 1.69% at 20 years; 2.61% at 25 years [2, 54–57]	Very limited data
EIMs in UC	≈24%–25% long-term [49, 58, 59] - PSC ≈2%–5% [39, 60] - Skin 9% [40] - Joint 8.9% at 10 years [36]	Similar magnitude; limited pediatric-only data [41].

Reported cumulative risks of major disease progression outcomes in Crohn’s disease (CD) and ulcerative colitis (UC) in adult- and pediatric-onset populations, including historical and contemporary estimates when available. Values are presented as approximate cumulative incidence at different time points. Historical data generally reflect pre-biologic or early biologic eras, whereas contemporary data reflect more recent therapeutic periods. When specified, very early-onset IBD (VEOIBD) is shown separately. Data in pediatric cohorts are limited for several outcomes, particularly colorectal cancer (CRC) and extraintestinal manifestations (EIMs). B1–B2–B3, Montreal behavior classification (inflammatory, stricturing, penetrating); CD, Crohn’s disease; CRC, colorectal cancer; EIMs, extraintestinal manifestations; PSC, primary sclerosing cholangitis; RR, relative risk; UC, ulcerative colitis; VEOIBD, very early-onset inflammatory bowel disease.

In UC, the dominant pattern of structural progression is proximal extension of colitis among patients with limited disease at diagnosis. Proximal extension estimates are supported by both population-based inception cohorts and pooled evidence from systematic reviews/meta-analyses. A Danish population-based inception cohort found that about one third of patients with proctitis or left-sided colitis extended to pancolitis (E3) within seven years [62]. A systematic review of 17 population-based UC cohorts reported pooled rates of progression from proctitis to left-sided disease in around 28%–30%, proctitis to extensive in 14%–16%, and left-sided to extensive in 21%–34%, with an overall 5-year cumulative progression risk of about 13% (Table 1) [42].

Estimates for EIMs are derived from population-based inception cohorts, large registries (e.g. ENEIDA), and systematic reviews/meta-analyses, which contributes to variability in reported incidence and prevalence. EIMs occurred in 20%–35% of IBD patients across long-term cohorts [36, 63–65], often associating with more extensive colonic involvement and markers of systemic inflammation such as elevated C-reactive protein (Table 1) [66].

**Figure 1:**
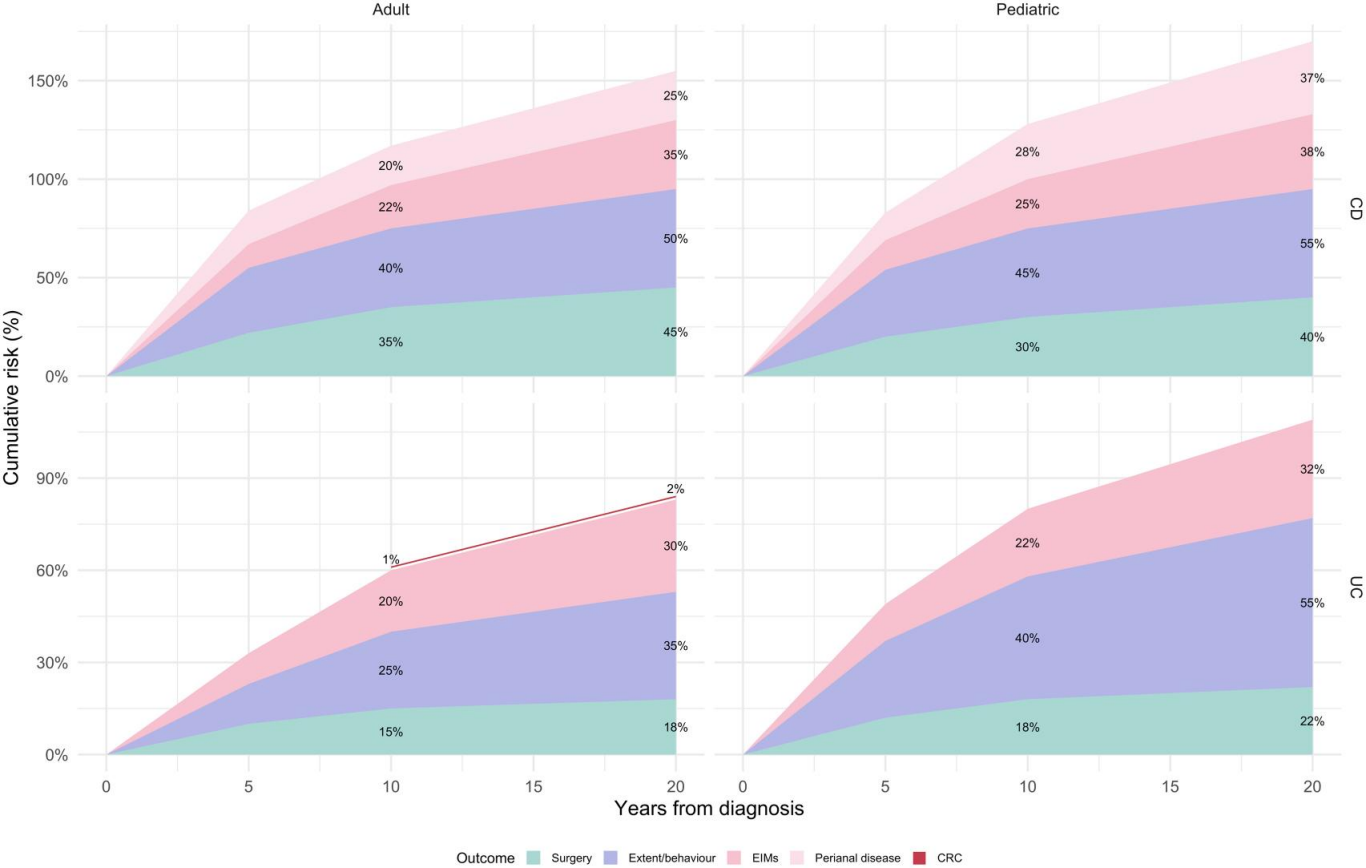
Cumulative risk of disease progression over time in adult and pediatric inflammatory bowel disease. Stacked area plots show the cumulative incidence (%) of different disease progression outcomes over 20 years from diagnosis in adults and children with Crohn’s disease (CD) and ulcerative colitis (UC). Outcomes include surgery, extent/behavior progression, extraintestinal manifestations (EIMs), perianal disease, and colorectal cancer (CRC). Percentages represent cumulative incidence for each outcome independently and are not additive, as individual patients may experience more than one type of progression during follow-up. Values displayed within each area correspond to the estimated cumulative risk at specific time points.

Surgical risks are informed by both long-running historical population-based inception cohorts and contemporary pooled estimates from meta-analyses of population-based cohorts. Contemporary population-based studies report lower cumulative risks of intestinal resection/colectomy than historical cohorts [47, 54]. Regarding colorectal neoplasia, a historic meta-analysis in UC suggested cumulative CRC risks of 2%, 8%, and 18% at 10, 20, and 30 years of disease duration, respectively. More contemporary pooled data, however, indicate substantially lower absolute risks of approximately 1.15% at 15 years, 1.69% at 20 years and 2.61% at 25 years, reflecting improved surveillance and inflammation control (Table 1 and Fig. 1).

### Crohn’s disease

#### Phenotype progression and perianal disease

Multiple population-based cohorts and inception studies converge on the finding that CD frequently progresses from an inflammatory to a complicated phenotype over time. In aggregate data, approximately 33% of adult patients’ progress from B1 to B2/B3 within 5 years, and around 50% do so within 20 years [1, 5, 9]. The Epi-IBD inception cohort, which enrolled incident IBD cases across 29 European centers, reported that 14% of patients with inflammatory CD at baseline developed stricturing or penetrating behavior within the first five years after diagnosis [5]. These data are comparable to the Hungarian cohort [10]. Older data dating back to the 1990s report a cumulative probability of developing B2/B3 of 66% at 15 years-time (Fig. 1) [11].

Specifically, the Olmsted County cohort found cumulative risks of 33.7% at 5 years and 50.8% at 20 years for developing stricturing or penetrating complications after diagnosis [9]. Other cohorts report similar rates, with 37%–39% at 10 years and 54%–78% at 25–30 years [67]. The risk is highest in patients with ileal or ileocolonic disease, younger age at diagnosis, extensive luminal involvement, and perianal disease [68].

Change in disease location is less common but clinically relevant. In the Veszprém province cohort, change in location was observed in roughly 9% of patients and was associated with smoking, whereas age at diagnosis did not significantly influence location change [12]. A previous study highlighted different genetic background depending on CD location and this could explain the lack of change in disease distribution observed, as compared with other disease behavior patterns [1].

Importantly, a subset of patients initially present with a seemingly mild disease course. A recent analysis of 2,315 individuals with mild IBD at diagnosis showed that 24.5% of those with UC and 46% of those with CD progressed to moderate–severe disease during follow-up. Progression was defined as the need of advanced therapy, immunosuppressants, hospitalization, surgery or new perianal disease whatever occurred first. Notably, pediatric onset had the highest likelihood of progression [13]. These findings reinforce that a mild phenotype at diagnosis does not guarantee long-term stability.

It is crucial to note that age at onset is a key determinant of this progression. Pediatric-onset CD exhibits a more aggressive phenotype [14]. Comparative studies suggest that pediatric CD has substantially higher behavior progression rates than adult-onset CD, with an incidence of progression from inflammatory to complicated (B2/B3) that may reach ≈34% in pediatric CD vs ≈16% in adult CD during follow-up (Fig. 1) [69–72].

Furthermore, a classic complication of CD is the onset of perianal disease, which is present at diagnosis in 11%–19% of patients and rises to 18%–20% at 10 years according to recent population-based cohort studies and meta-analyses [27–30]. The cumulative risk of developing perianal fistula is higher in pediatric-onset Crohn’s disease, reaching 28% at 10 years and 45% at 30 years [31, 72].

Importantly, recent cohorts show a decline in the cumulative 10-year risk of perianal fistula (falling from 24% to 12% between older and newer eras), indicating a positive effect of therapeutic advancements and early biologic therapy [10, 26].

#### Need for surgery

Need for surgical intervention is commonly used as a proxy for disease progression. Surgical rates have shown a decline in recent decades, a finding that underscores the disease-modifying impact of contemporary therapy and early treatment strategies.

In historical CD cohorts (e.g. Danish cohorts from 1962 to 1987), intestinal resection was common, with cumulative rates reaching 61% at 10 years and 82% at 20 years [15]. However, more recent population-based cohorts (reflecting the era of biologics and earlier immunomodulator use) show a significant reduction. Contemporary 5-year intestinal resection rates are estimated between ≈18% and ≈35%. Although this reduction is evident, surgery remains frequent in CD over long-term follow-up [16, 17, 73, 74].

In Epi-IBD, 22% of CD patients required intestinal resection within five years, despite widespread use of immunomodulators and biologics [5]. Early bowel resection rates in pediatric CD cohorts are generally lower than those reported in historic adult cohorts, but are still substantial, with 5-year cumulative rates typically ranging from 13% to 34% [18–21, 75]. In very early-onset inflammatory bowel disease (VEOIBD) cohorts, the reported rates of bowel surgery in CD are approximately 3% at 1 year, 12% at 3 years, and 15% at 5 years [22]. These rates are lower than those seen in older pediatric populations and adult-onset disease. Other multicenter studies of VEOIBD and infantile IBD confirm a low cumulative risk of surgical intervention (typically <15% at 5 years), though patients with monogenic IBD have higher rates of surgery [23, 58].

Predictors of surgery in CD consistently include ileal/ileocolonic location, stricturing/penetrating behavior, and smoking [76]. In addition, studies comparing sporadic vs familiar IBD have reported increased risk of surgery in patients with family history of IBD [77]. Where reported, overall surgical rates have declined over time; however, most population-based cohorts and registries do not provide consistent granularity on surgical indications (e.g. obstruction/stenosis vs perforation vs inflammatory complications) or urgency, limiting inference on whether the drivers of surgery have shifted across therapeutic eras.

#### Extra intestinal manifestations

Long-term mixed IBD cohorts, including CD and UC, report EIMs in roughly 24% of patients over median follow-up periods of around 14 years, with higher rates in CD (35%) than UC (27%) [63]. In CD, these manifestations often cluster with more extensive or aggressive disease and may contribute importantly to disability [37, 38].

EIMs are a clinically significant component of CD and frequently reflect the systemic nature of chronic inflammation. In the European population-based inception cohort by Isene *et al.*, which followed 1,145 newly diagnosed IBD patients for a median of 10.1 years, the cumulative prevalence of a first EIM was 16.9%, with a significantly higher burden in CD than in UC [36]. Specifically, 20.1% of patients with CD developed immune-mediated EIMs (articular, ocular, cutaneous, hepatobiliary), compared with 10.4% of those with UC (*P *< 0.001) [36]. Peripheral arthritis was the most common manifestation, affecting 12.9% of patients with CD vs 8.1% in UC (*P *= 0.01) [36]. These findings are consistent with other prospective cohorts showing that EIMs tend to cluster in patients with colonic or ileocolonic involvement, higher inflammatory burden, and younger age at diagnosis [38]. Earlier studies have reported even higher rates, reflecting differences in study design and criteria, but consistently underscore that CD carries a greater systemic inflammatory load than UC [40]. In the Swiss cohort that studied 3,298 patients found that 48.8% of patients with IBD suffered from arthritis/arthralgia during their disease course, they found female gender, disease duration, surgery, presence of other EIM and treatment with anti-TNF to be risk factors for the onset of joint manifestations in CD and UC/IBD-unclassified (IBDU) patients [40]. Furthermore, data from this same cohort showed that skin manifestations occurred more frequently in CD (19.2%) vs UC (9.5%), in female and in those with positive family history of IBD [40]. One of the largest cohorts including 12,083 European IBD patients identified EIMs being more frequent in CD, female, those needing surgery and smokers (excluding for risk of primary sclerosing cholangitis [PSC]). In this study, contrary to other studies, only 2% of patients had multiple EIMs.

#### Dysplasia and colon cancer

CRC in IBD arises primarily as a consequence of chronic, longstanding mucosal inflammation of the colon, which promotes genomic instability, oxidative stress, and accelerated epithelial turnover [32]. Unlike sporadic CRC, where the adenoma–carcinoma sequence predominates, IBD-associated CRC often follows an inflammation–dysplasia–carcinoma pathway, characterized by flat, multifocal, and rapidly progressive dysplasia [33]. The risk is modulated by several well-established factors: extent of colonic involvement, duration of disease, cumulative inflammatory burden, presence of strictures, coexistent PSC, family history of CRC, and early age at onset [34]. In CD, cancer risk is particularly heterogeneous and depends on whether there is chronic colonic inflammation and the extent of it, repeated flares, persistent histologic activity, or longstanding stricturing segments.

CD is associated with an increased risk of small-bowel adenocarcinoma compared with the general population, but this occurs in a very low number of patients [35, 78], however, when colonic involvement is present, absolute risk of colitis-associated CRC especially those with more than 50% of the colon affected for over ten years, have a significantly increased risk, similar to that seen in ulcerative colitis [34, 79]. Nonetheless, the absolute CRC incidence in colonic CD remains low compared with UC, and is further mitigated in contemporary cohorts by surveillance and improved inflammatory control.

### Ulcerative colitis

#### Proximal extension

Extension of disease is a key risk factor, as extensive disease at diagnosis is linked to a higher risk of colectomy and systemic complications. In UC, the main form of progression is the proximal extension of inflammation [43]. Patients initially diagnosed with proctitis (E1) or left-sided colitis (E2) face a cumulative risk of inflammation extending proximally to reach extensive colitis (E3). In the Danish population-based inception cohort of UC diagnosed in 2003–2004, 73% of patients had limited disease (E1/E2) at diagnosis. Over at least seven years of follow-up, 33% of these patients developed proximal extension, with 23% progressing to extensive colitis (E3) and 10% from E1 to E2 (Fig. 1). Disease extent at diagnosis was the only independent predictor of extension to E3 [62].

Fumery’s systematic review of 17 population-based cohorts (over 15,000 UC patients) showed that at diagnosis, left-sided colitis represented around 40%, extensive colitis 30% and proctitis 29% of cases [44]. Progression rates from proctitis to left-sided disease were 28%–30%, from proctitis to extensive 14%–16% and from left-sided to extensive 21%–34%, with an overall cumulative 5-year progression risk of approximately 13%. Interestingly, these extension rates appeared relatively stable across regions and eras, including pre-biologic and biologic periods.

Pediatric UC typically presents more extensively than adult UC [24, 45]. In addition, the risk of proximal extension is substantial: among children with proctitis or left-sided colitis at diagnosis, 30%–49% experience extension over 5–10 years, and this risk is similar to or higher than in adults [24, 45, 46].

#### Colectomy

Total colectomy is the most definitive outcome for UC patients, reserved for refractory disease, toxic megacolon, or neoplasia prevention [2]. Similar to CD, colectomy rates have substantially declined. While historical 10-year rates approached 24% [47], pooled contemporary meta-analytic estimates report a cumulative incidence of ≈4.4% at 1 year, ≈10.1% at 5 years, and ≈14.6% at 10 years in adults [17]. Nonetheless, colectomy rates also vary depending on geography, likely due to the differences in the epidemiological stage of IBD incidence and prevalence [80], as an example, Korean data from 1986 to 2015 showed a 20-year risk of colectomy for newly diagnosed UC of 5.1% (Fig. 1) [81]. In the Epi-IBD UC cohort, despite more aggressive use of immunomodulators and biologics than in earlier decades, colectomy rates over the first five years were not substantially lower, although immunomodulator use was associated with reduced hospitalization [6, 7].

Short-term colectomy rates (1 year) in pediatric UC are approximately 3%–6%, and medium-term rates (5 years) are 9%–14%; these rates have declined in the biologic era, with recent population-based cohorts reporting 5-year colectomy rates of 9%–13% [49, 51–53]. Very early-onset UC/IBD-U cohorts show similar patterns, with colectomy risks approaching 1%–14% at five years [58].

#### Extraintestinal manifestations

Although global EIMs appear to be more frequent in patients with CD, EIMs are not rare among UC patients with over a quarter of them experiencing at least one new EIM during follow-up. Extensive disease in UC is the primary predictor for developing EIMs, increasing the risk more than threefold (odds ratio [OR] ≈3.6) in those with extensive colitis [36, 59]. Reported EIM prevalence over 8–14 years of follow-up ranged widely from 8.9% to 66%, reflecting heterogeneity in definitions and ascertainment [65]. Some EIMs are more frequently reported among patients with UC than in CD, as PSC. Large meta-analyses and population-based studies consistently report a lifetime risk of PSC of approximately 2%–5%, with some variation by geography, diagnostic criteria, and extent of colitis (Table 1 and Fig. 1) [39]. The American College of Gastroenterology guideline states the prevalence is about 5%, with higher risk in men and those with pancolitis [60]. On the other hand, PSC risk is significantly lower in CD typically <1% [39, 82].

#### Colorectal cancer and dysplasia

Historical data suggested high cumulative CRC risks, but more recent meta-analyses indicate a decline in absolute risk. The current cumulative risks of CRC in adults with UC are approximately 0.8% within 10 years, 2.2% within 10–20 years, and 4.5% after 20 years of disease duration. These risks are higher in patients with long-standing, extensive colitis, with standardized incidence ratios approaching 4.0–4.8 compared with the general population, and are further increased in those with persistent histologic inflammation or a family history of CRC and those with coexistent PSC-IBD [2, 55]. However, due to improved endoscopic surveillance and better inflammation control, the cumulative CRC incidence in contemporary cohorts has declined [2, 56]. Recent estimates by Stidham and Higgins [32, 57] show cumulative rates of approximately 1.15% at 15 years, 1.69% at 20 years, and 2.61% at 25 years in adults, much lower than the classic 2%/8%/18% at 10/20/30 years described by Eaden *et al.* [57].

Recent cohort data suggest that biologic exposure may be independently protective against colorectal stricture development, plausibly through improved inflammation control and prevention of chronic injury leading to fibrotic complications [83]. Pseudopolyps are considered sequelae of repeated inflammation-healing cycles, and their occurrence is more closely linked to cumulative mucosal inflammatory burden than to biologic therapy. In addition, prior biologic or immunosuppressant exposure has been inversely associated with dysplasia in pseudopolyp-like lesions, suggesting a potential protective effect on neoplastic transformation [84].

This decline is likely multifactorial. It is attributed to improved long-term inflammation control with maintenance therapy (5-ASA, thiopurines, anti-TNF, and anti-integrin), alongside advances in surveillance quality and strategy, including high-definition endoscopy, chromoendoscopy, and earlier and more frequent endoscopic resection of visible dysplasia [34]. However, the relative contribution of surveillance versus inflammation control cannot be reliably quantified from observational cohort data and secular changes in other CRC risk modifiers (e.g. smoking patterns, diet, and obesity) may also contribute and confound temporal comparisons. Modern surveillance may additionally shift outcomes towards earlier detection and endoscopic management of intermediate endpoints such as dysplasia, thereby preventing progression to CRC. However, pseudopolyps and dysplasia detection rates are not consistently reported across population-based cohorts using harmonized definitions, limiting quantitative comparisons over time. Nonetheless, CRC risk remains concentrated in patients with long-standing, extensive colitis, especially those with concomitant primary sclerosing cholangitis or a family history of CRC.

### Determinants and modifiers of progression

Progression is not random; it is strongly influenced by intrinsic disease features and treatment exposure [85]. Intrinsic factors such as ileal/ileocolonic location, pediatric onset, perianal disease and need for systemic steroids showing an initial complicated behavior in CD are the most robust predictors of future adverse outcomes and surgery, whereas colonic disease appears protective against surgery [76, 86, 87]. Smoking acts as a disease modifier, associated with CD progression (including location change) and a higher surgery risk [86]. In UC, more extensive disease at diagnosis predicts both proximal extension and colectomy, and progression from limited to extensive colitis further increases colectomy risk [2, 88]. Elevated C-reactive protein and the presence of EIMs cluster with extensive disease, reinforcing the notion that systemic inflammatory burden is a marker of more severe course.

Across both diseases, degree of inflammatory control is central to long-term outcomes [89]. Multiple reviews and cohorts highlight mucosal healing as a key correlate of reduced hospitalization, colectomy and relapse in UC, and likely as a proxy for reduced structural progression in CD, although high-quality prospective disease-modification trials are still needed [89, 90]. Contemporary treat-to-target strategies, formalized through the STRIDE-II consensus, emphasize deep remission targets, including mucosal healing and although not formal targets it also recognize the prognostic impact of histologic healing in UC and transmural healing in CD [91]. Importantly, these therapeutic advances provide a plausible explanation for the most consistent temporal improvements observed in population-based outcomes over recent decades. Therapy and era effects appear most evident for hard outcomes such as surgery and hospitalization. Early and consistent use of immunomodulators and biologics is associated with lower risks of surgery and hospitalization in observational cohorts. Biologics (hazard ratio = 0.66–0.72) and thiopurines (hazard ratio = 0.62–0.81) were independently protective against abdominal surgery in CD [92, 93]. Moreover, biologic exposure was associated with a lower risk of location change in CD (hazard ratio ≈0.3) [94]. Achieving endoscopic mucosal healing within the first year, is a particularly strong prognostic factor, associated with an approximately 78% reduction in colectomy risk in UC (relative risk ≈0.2). These findings support the “treat-to-target” strategy in IBD management [91].

In addition to this, cumulative inflammatory burden is increasingly recognized as a key determinant of long-term complications including CRC. Previous studies showed that time-adjusted cumulative inflammation was associated with CRC in UC [54, 57].

Nevertheless, the extent to which modern therapies modify short-term structural progression remains uncertain. Some large population-based studies suggest that 5-year behavior progression and surgery rates have not dramatically fallen compared with cohorts from earlier decades, indicating that the impact of current strategies on certain hard structural endpoints may be modest over this time horizon and may depend on very early intervention before irreversible tissue remodeling occurs.

Future evidence to show the impact of broader access to biologic therapy, partly facilitated by the increasing availability of biosimilars and evolving reimbursement policies, would potentially contribute to a better understanding of the observed temporal improvements in hard outcomes.

Looking forward, emerging therapeutic targets may further shift progression patterns, particularly if they can address pathways beyond inflammation alone. For example, anti-TL1A agents are being explored as potentially disease-modifying therapies, and preclinical data suggest effects on immune–stromal interactions that could be relevant to intestinal tissue remodeling. Whether such approaches translate into durable prevention of fibrostenotic complications in humans remains to be demonstrated in prospective trials with structural endpoints.

## Discussion

This structured review summarizes contemporary evidence on disease progression in CD and UC, focusing on phenotype or extent evolution, surgery, EIMs, and colorectal neoplasia. The picture that emerges is of two chronic, heterogeneous conditions with substantial long-term risks, albeit with notable differences both between CD and UC, and between adults and children.

In CD, progression from inflammatory to stricturing or penetrating behavior remains common despite therapeutic advances, with roughly one third of adults progressing within five years and about half over longer horizons. Perianal disease and need for intestinal resection continue to affect a sizeable minority of patients. Pediatric CD demonstrates at least comparable, and in some analyses more aggressive, behavior progression, underscoring the need for early risk stratification and careful monitoring in younger patients.

In UC, proximal extension from limited disease to more extensive colitis occurs in about one third of patients over the first decade, and colectomy remains necessary in a non-negligible proportion, particularly among pediatric patients. At the same time, contemporary data suggest that colectomy rates and, especially, CRC incidence have declined in modern cohorts. This improvement likely reflects a combination of better inflammation control, structured surveillance, high-definition and chromoendoscopy, and more active endoscopic management of dysplasia.

Across both diseases, EIMs occur in about a quarter of patients over long-term follow-up, often clustering with extensive or more aggressive disease and contributing significantly to morbidity. The strong associations between extent, systemic inflammatory burden and EIMs suggest that deep and sustained control of inflammation is critical not only for intestinal outcomes but also for systemic ones.

Of note, the clearest temporal improvement is seen in hard outcomes such as surgery and CRC risk. In CD, intestinal resection rates in historical cohorts reached 61% at 10 years and 82% at 20 years [15, 17], whereas contemporary cohorts report 18%–35% at 5 years (including 22% at 5 years in Epi-IBD) [5]. In UC, 10-year colectomy risk has declined from historical estimates of approximately 24% [46], to contemporary pooled estimates of approximately 14.6% [48]. Likewise, CRC risk in UC has fallen from classic estimates of 2%/8%/18% at 10/20/30 years [57], to approximately 1.15% at 15 years, 1.69% at 20 years, and 2.61% at 25 years in modern analyses [56]. In contrast, behavior progression in CD and proximal extension in UC remain frequent, and pediatric-onset disease tends to follow a more extensive or progressive trajectory than adult-onset IBD, reinforcing the need for early risk stratification.

Several consistent predictors of progression can be incorporated into clinical decision-making. In CD, ileal or ileocolonic disease, perianal involvement, smoking and the need for systemic steroids are strong markers of a more aggressive course and higher surgery risk. In UC, extensive colitis at diagnosis and subsequent extension are key risk factors for colectomy and CRC. These factors, together with age at onset and comorbidities such as PSC, should inform risk stratification and timing of advanced therapies.

The question of whether we can truly modify the natural course of IBD remains incompletely answered. Observational cohorts and reviews suggest that early and sustained use of immunomodulators and biologics is associated with reduced hospitalization and surgery, and that mucosal healing correlates with better long-term outcomes. However, some population-based studies note that 5-year rates of behavior progression and surgery have not dramatically fallen compared with cohorts from previous decades, indicating that the impact of current therapies on hard structural endpoints may be modest at this time scale. High-quality prospective “disease modification” trials, with structural progression and surgery as primary outcomes, are still needed.

Despite the strengths of focusing on population-based inception cohorts and large registries, several limitations should be acknowledged. First, definitions of “progression” vary across studies (e.g. behavior change vs complications vs treatment escalation), and follow-up duration and censoring strategies differ, limiting direct comparability of absolute estimates. Second, ascertainment of outcomes has evolved over time (wider use of cross-sectional imaging, intestinal ultrasound, fecal calprotectin monitoring, high-definition endoscopy, and chromoendoscopy), which may influence detection of complications and dysplasia and introduce surveillance-related bias. Third, treatment era comparisons are inherently observational and may be confounded by indication and secular trends in care pathways, making causal inference regarding disease modification challenging. Fourth, key outcomes such as indications for surgery (e.g. inflammatory complications vs fibrostenotic obstruction) and dysplasia detection rates are inconsistently reported across cohorts, preventing robust synthesis of temporal changes in these more granular endpoints. Finally, most available evidence is derived from European and North American cohorts from large tertiary hospitals, and generalizability to regions with emerging IBD epidemiology or smaller hospitals with different access to advanced therapies may be limited. Future prospective studies and pragmatic disease-modification trials with harmonized outcome definitions, including structural endpoints, indication-specific surgery, and dysplasia detection, are needed to clarify how modern strategies can durably alter the natural history of IBD.

Knowledge gaps persist, particularly concerning the need for robust population-based estimates of the incidence of new EIMs stratified by age and disease type, and quantitative data on pediatric dysplasia/CRC incidence. Ultimately, prospective disease-modification trials are needed to definitively establish the causal impact of the timing of advanced therapies on structural progression and long-term hard outcomes.

## Conclusion

Population-based and inception cohorts, together with systematic reviews, provide a coherent picture of IBD as a chronic, progressive condition in a substantial subset of patients. In CD, structural complications and surgery remain common, particularly among patients with ileal or perianal disease, in those who smoke and in pediatric CD. UC commonly undergoes proximal extension, and colectomy risk, while lower than historically reported, continues to accumulate, especially in pediatric cohorts.

Crucially, the data indicate a measurable era effect, with declining surgical rates in both CD and UC, and lower CRC incidence in UC, correlating with earlier and broader adoption of immunomodulators and biologics, and improved surveillance. These findings reinforce that therapeutic strategies aiming for mucosal healing and sustained inflammatory control are critical for mitigating the long-term, progressive course of IBD.

## Supplementary Material

goag013_Supplementary_Data
