# Supplementary material for: Global patterns of disease progression in inflammatory bowel disease: a comprehensive synthesis of contemporary population-based cohorts
Source: Gastroenterol Rep (Oxf). 2026 Feb 20;14:goag013. doi: 10.1093/gastro/goag013 (PMC12921713; doi:10.1093/gastro/goag013)
Supplement: goag013_Supplementary_Data [file goag013_supplementary_data.docx]

**Supplementary Material**

1. Search Strategy

Last search 7^th^ September 2025

Databases: PubMed, EMBASE

- Search terms: combination of MeSH and free-text terms, including:
- ( "Inflammatory Bowel Diseases"[Mesh] OR "Colitis, Ulcerative"[Mesh] OR "Crohn Disease"[Mesh] OR IBD[tiab] OR "inflammatory bowel disease*"[tiab] OR UC[tiab] OR "ulcerative colitis"[tiab] OR CD[tiab] OR "Crohn* disease"[tiab] ) AND ( "Disease Progression"[Mesh] OR progression[tiab] OR worsening[tiab] OR "disease course"[tiab] OR "disease trajectory"[tiab] ) AND ( "disease extent"[tiab] OR extension[tiab] OR "disease distribution"[tiab] OR "proximal spread"[tiab] OR "Extraintestinal Manifestations"[Mesh] OR "extraintestinal manifestation*"[tiab] OR EIM*[tiab] OR IMID*[tiab] OR "Arthritis"[Mesh] OR "Spondylarthropathies"[Mesh] OR "Primary Sclerosing Cholangitis"[Mesh] OR "Uveitis"[Mesh] OR "Erythema Nodosum"[Mesh] OR "Pyoderma Gangrenosum"[Mesh] OR "Colorectal Surgery"[Mesh] OR "Colectomy"[Mesh] OR "Proctocolectomy, Restorative"[Mesh] OR "Intestinal Resection"[Mesh] OR surgery[tiab] OR resection[tiab] OR colectomy[tiab] OR proctocolectomy[tiab] OR stoma[tiab] OR ostomy[tiab] OR "Colorectal Neoplasms"[Mesh] OR "Colitis-Associated Neoplasms"[Mesh] OR "Precancerous Conditions"[Mesh] OR "Dysplasia"[Mesh] OR "colitis-associated cancer"[tiab] OR "IBD-associated neoplasia"[tiab] OR dysplasia[tiab] OR cancer[tiab] )
- No language restrictions.
- Hand-searching of references and citation tracking of included studies.
